# Nicotinic Acetylcholine Receptors Modulate T-Tubule Architecture Under Sympathetic Overstimulation

**DOI:** 10.3390/ijms27219597

**Published:** 2026-10-27

**Authors:** Cai He, Qi Zeng, Junwei Zhao, Yang Meng, Yingna Guo, Hongguang Wang, Shi-Qiang Wang, Xiang Li, Tingting Hou

**Affiliations:** 1 Beijing Life Science Academy, Beijing 102209, Chinawsq@pku.edu.cn (S.-Q.W.); 2 State Key Laboratory of Membrane Biology, College of Life Sciences, Peking University, Beijing 100871, China

**Keywords:** nicotine, acetylcholine receptor, cardiomyocyte, transverse tubule, cardiomyocyte morphology

## Abstract

Pathological remodeling of transverse tubules (T-tubules, TTs) is commonly observed in cardiac disease and may be promoted by sustained adrenergic stimulation. Although cholinergic signaling has been associated with cardioprotective effects, it remains unclear whether it directly modulates TT integrity in cardiomyocytes. Here, we examined how cholinergic receptor activation affects TT organization during acute β-adrenergic stimulation in isolated adult rat cardiomyocytes. Cells were exposed to isoproterenol (ISO), alone or in combination with acetylcholine (ACh) or nicotine (Nic). Confocal imaging showed that ISO disrupted TT organization and reduced the cellular length-to-width (L/W) ratio. ACh or Nic alone altered TT architecture, primarily by reducing TT power and increasing longitudinal tubule density; however, co-treatment with either agonist was associated with altered TT remodeling responses to ISO in TT structure and cell morphology. The muscarinic receptor agonist xanomeline (Xano) did not reproduce these effects. These results suggest a cardiomyocyte-autonomous role for nAChR signaling in limiting acute β-adrenergic stimulation-induced TT remodeling. Further studies are required to identify the nAChR subtype and downstream signaling mechanisms involved and to determine the relevance of these findings in vivo.

## 1. Introduction

The transverse tubules (T-tubules, TTs) of mammalian cardiomyocytes are invaginations of the surface membrane that markedly increase membrane surface area and transduce extracellular signals into the intracellular compartment. Pathological remodeling of the TT network, typically characterized by reduced overall density and loss of transverse orientation, is a hallmark of various cardiac diseases, including heart failure, chronic ischemia, and atrial fibrillation [1,2]. The formation and maintenance of this tubular system are highly dynamic, with its regulation by several proteins (e.g., bridging integrator 1, BIN1, and junctophilin 2, JPH2 [1,3]) and diverse factors. For instance, activation of the β-adrenergic pathway, which mediates the positive inotropic effect of sympathetic stimulation in part by increasing voltage-gated Ca^2+^ current [4,5,6,7] can destabilize TT structure. This occurs through disrupted BIN1 microdomains [8] and increased calpain-mediated JPH2 cleavage [9,10,11]. In physiological contexts, sympathetic actions are counterbalanced by the parasympathetic cholinergic system. Conventionally, acetylcholine (ACh) derived from either vagal nerve endings or cardiomyocytes themselves binds to M2 muscarinic acetylcholine receptors (mAChR), which are the predominant cardiac ACh receptor, thereby exerting negative chronotropic, dromotropic, and inotropic effects [12]. The activation of ACh receptors has been increasingly recognized for its significant protective effect on the heart [13,14]. Most studies on this cardioprotection have focused on immune modulation via M2 receptor signaling [15]. In recent years, expression of nicotine acetylcholine receptors (nAChR), especially α7nAChR, has been identified on ventricular cardiomyocytes [16]. Moreover, previous studies reported that cardiomyocytes lacking α7nAChR were intrinsically more susceptible to cytotoxicity induced by the non-selective β-adrenergic receptor agonist isoproterenol (ISO), indicating that α7nAChR exerts a cell-autonomous protective role beyond its anti-inflammatory function [17]. Despite these insights, the structural basis of this non-inflammatory cardioprotection remains obscure, particularly regarding the preservation of TTs microdomains. Therefore, determining whether and how cholinergic signaling mitigates TT degradation under sympathetic stress is critical. Here, we used isolated cardiomyocytes treated with ISO, ACh, and specific cholinergic agonists/antagonists to assess TTs remodeling and uncover the underlying cell-autonomous protective mechanisms.

## 2. Results

### 2.1. ISO Induces Marked TT Disorganization in Isolated Adult Rat Cardiomyocytes

**Figure 1:**
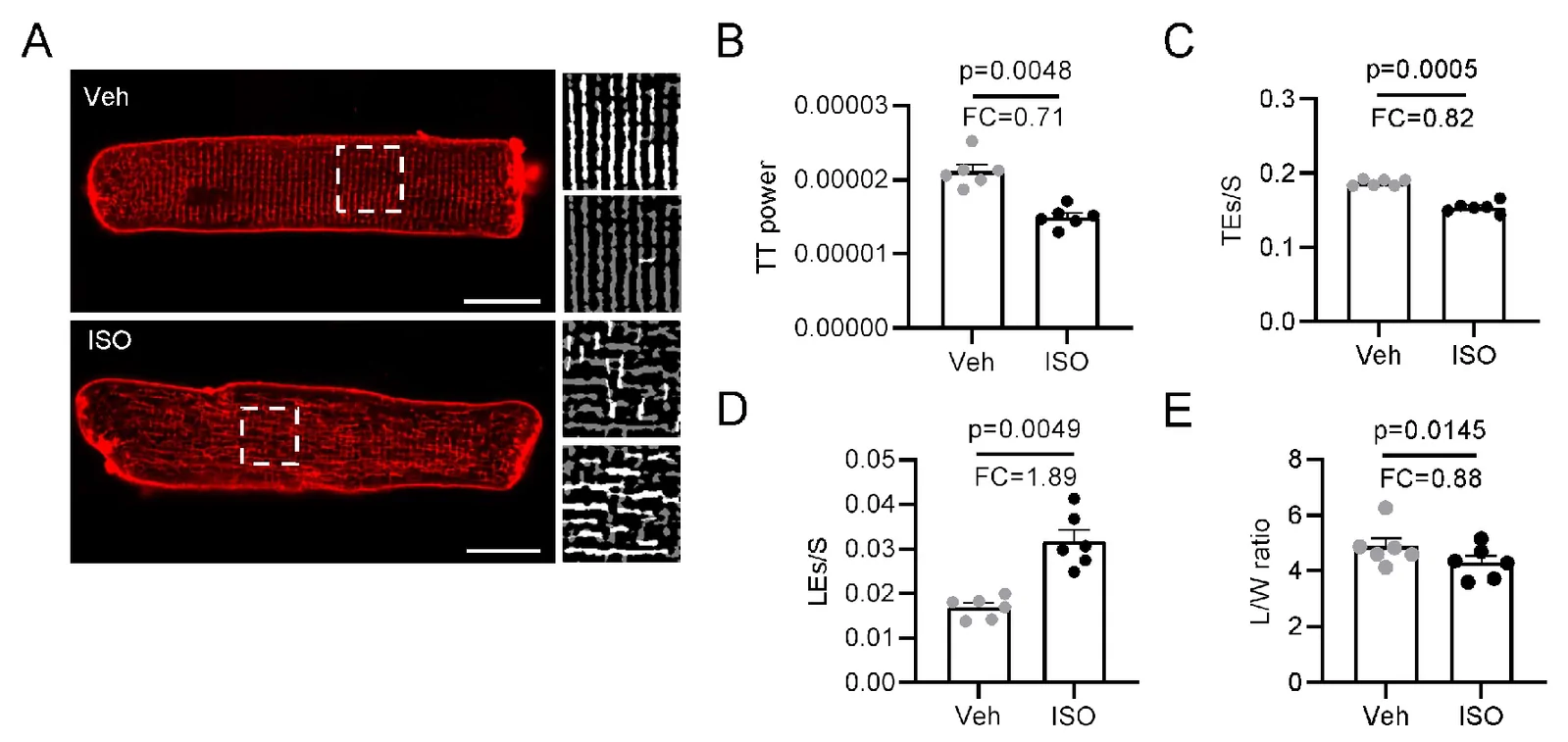
Effects of β-adrenergic agonists on transverse tubule (TT) organization in isolated adult rat cardiomyocytes. (**A**) Representative images of CellMask Deep Red-stained adult rat ventricular cardiomyocytes. The dashed white boxes indicate the regions of interest (ROIs) that are magnified on the right. The magnified panels show the skeletonized images identifying transverse elements (TEs—top) and longitudinal elements (LEs—bottom) of the TT network. Veh—Vehicle, ISO—isoproterenol, 100 nM for 48 h. Scale bar = 20 μm. (**B**–**D**) Quantitative analysis of the TT network structure: TT power, derived from Fast Fourier Transform (FFT) analysis, reflecting the regularity of the TT network (shown in panel (**B**)), the quantification of transverse elements normalized to the area of ROI (TEs/S, shown in panel (**C**)), as well as longitudinal elements normalized to the area of ROI (LEs/S, shown in panel (**D**)). Data are presented as mean ± SEM. N = 6 rats (5 males and 1 female). (**E**) The length-to-width (L/W) ratio of cardiomyocytes in response to ISO. Data are presented as mean ± SEM. N = 6 rats (5 male and 1 female). Paired *t*-tests were used to compare Veh versus ISO within the same animals in (**B**–**E**). Exact *p*-values and fold change (FC) are shown above each figure. The 95% confidence intervals are provided in Supplementary Table S1.

To investigate the direct, cell-autonomous impact of sympathetic overstimulation on TTs architecture, we first treated primary adult rat cardiomyocytes with ISO for 48 h. ISO induced a pronounced derangement of TTs morphology (Figure 1A), characterized by a loss of transverse periodicity, an increase in longitudinal TTs, and the formation of sparse, fragmented tubule structures (Figure 1B–D). Concurrently, the length-to-width ratio (L/W ratio) was significantly decreased in the ISO group (Figure 1E), suggesting morphological deterioration of individual cells alongside possible functional decline.

### 2.2. Effects of Cholinergic Receptor Stimulation on TT Organization

**Figure 2:**
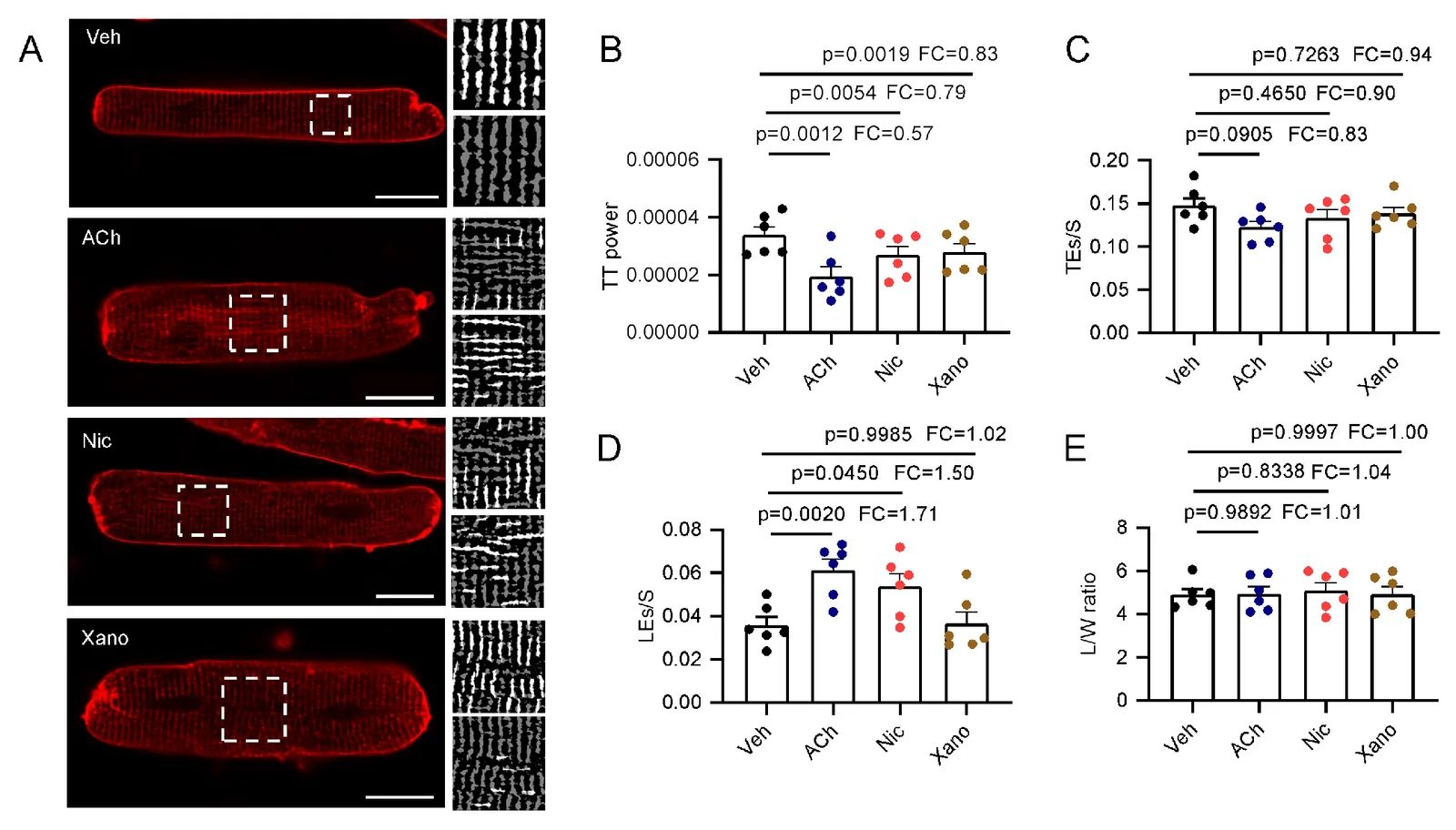
Effects of cholinergic receptor stimulation on TT organization. (**A**) Representative images of CellMask Deep Red-stained adult rat ventricular cardiomyocytes, as shown in Figure 1A. Ach—acetylcholine, 10 μM for 48 h; Nic—nicotine, 10 μM for 48 h; Xano—xanomeline, 10 μM for 48 h. Scale bar = 20 μm. (**B**–**D**) Quantitative analysis of the TT network structure as in Figure 1B–D. (**E**) The L/W ratio of cardiomyocytes in response to ACh, Nic or Xano. For (**B**−**E**), data are expressed as mean ± SEM. N = 6 rats (5 males and 1 female). Statistical significance was assessed by a repeated-measures (RM) one-way ANOVA with Tukey’s post hoc test. Exact *p*-values and fold change (FC) are shown above each figure. The 95% confidence intervals are provided in Supplementary Table S1.

We next asked whether cholinergic agonists could induce TT remodeling. To this end, we challenged the cells with acetylcholine (ACh, the endogenous ligand of both nAChRs and mAChRs), nicotine (Nic, nAChR agonist), or xanomeline (Xano, mAChR agonist) for 48 h. As shown in Figure 2B, all three agonists alone altered TT power relative to vehicle. In contrast, TEs/S was not significantly changed by ACh or Nic alone, although there was a trend toward a reduction with ACh and Nic; Xano did not significantly alter TEs/S either (Figure 2C). At the same time, ACh and Nic significantly increased LEs/S, whereas Xano did not (Figure 2D). None of the agonists changed the cell L/W ratio (Figure 2E). Together, these data suggest that even cholinergic signaling, and partial activation of nAChR alone, may influence TT dynamics, with a shift from transverse to longitudinal organization rather than a global loss of tubules.

### 2.3. Co-Activation of nAChRs by Acetylcholine or Nicotine Correlates with Attenuation of ISO-Induced T-Tubule Remodeling

**Figure 3:**
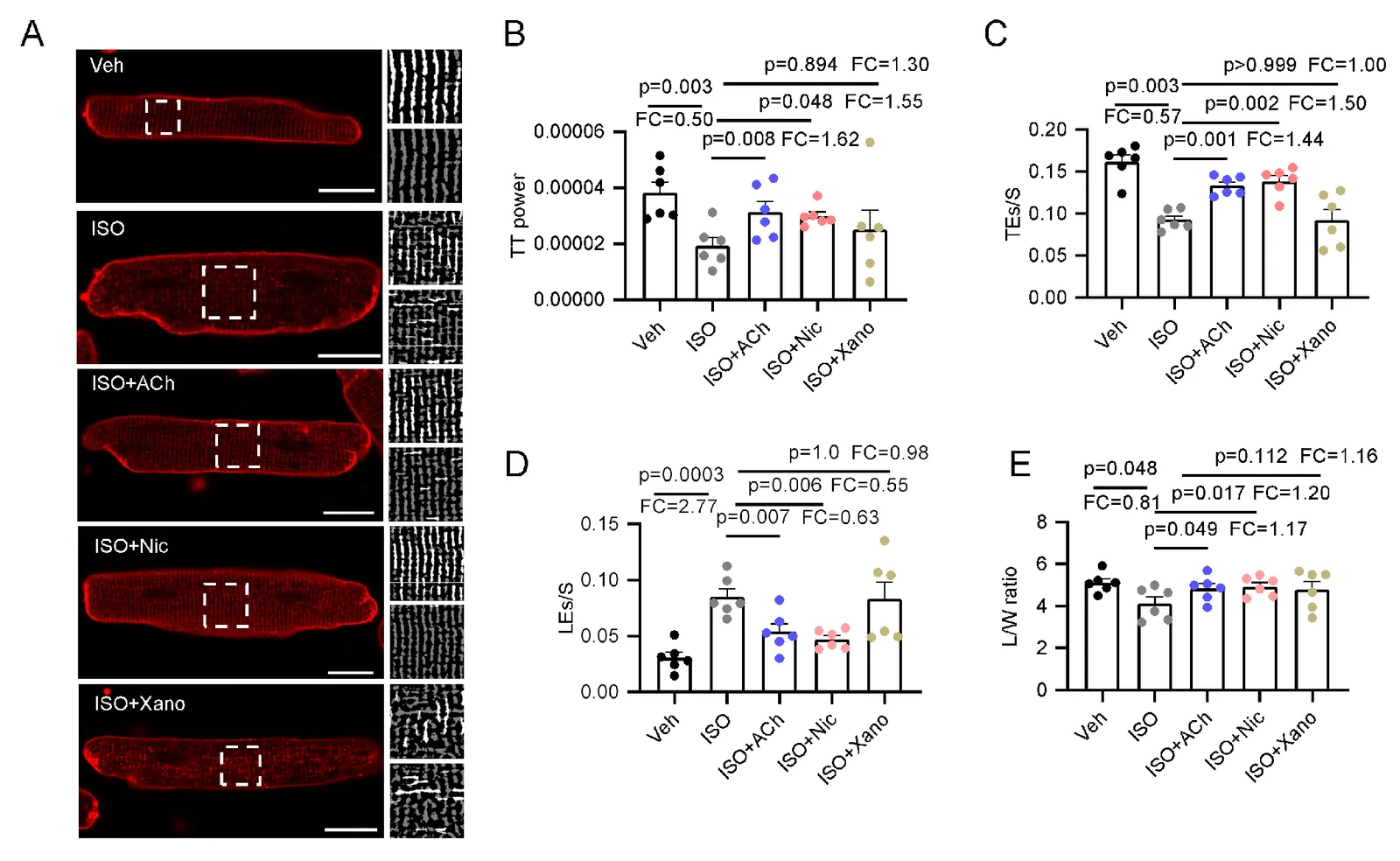
Co-administration of ISO with either ACh or Nic was associated with less pronounced ISO-induced TT structural changes. (**A**) Representative images of CellMask Deep Red-stained adult rat ventricular cardiomyocytes as shown in Figure 1A. ISO + ACh, ISO + Nic and ISO + Xano: co-treatment with isoproterenol (100 nM) and acetylcholine, nicotine, and xanomeline (10 μM each) for 48 h, respectively. Scale bar = 20 μm. (**B**–**D**) Quantitative analysis of the TT network structure as in Figure 1B–D. (**E**) The L/W ratio of cardiomyocytes in response to ISO, ISO + ACh, ISO + Nic, and ISO + Xano. Data are expressed as mean ± SEM. N = 6 rats (4 males and 2 females). Statistical significance was assessed by a RM one-way ANOVA with Dunnett’s post hoc test. Due to the high data dispersion observed in certain treatment groups (e.g., the ISO + Xano group in (**B**)), the assumption of sphericity was not required. Instead, the Geisser–Greenhouse correction was applied to robustly adjust the degrees of freedom and validate the ANOVA results. For clarity, only *p*-values for the pre-planned comparisons of interest are indicated above each figure. A complete table of exact *p*-values and 95% confidence intervals can be found in Supplementary Table S1.

Given that treatment with ACh or Nic alone produced modest TT morphological changes, we next evaluated their impact under sympathetic overstimulation. Notably, co-treatment of ISO with either ACh or Nic appeared to modulate T-tubule structural integrity, attenuating the ISO-induced disruption to varying degrees (Figure 3A). This effect was associated with increased density of transversally oriented elements and fewer disorganized tubule profiles (Figure 3B–D). Notably, this preservation was absent in the ISO + Xano co-treatment group. Additionally, the ISO-induced reduction in L/W ratio showed a tendency toward reversal upon co-treatment with ACh or Nic (Figure 3E), supporting a functional correlate of the observed structural preservation.

### 2.4. Pharmacological Evidence for nAChR-Mediated Attenuation of TT Remodeling

**Figure 4:**
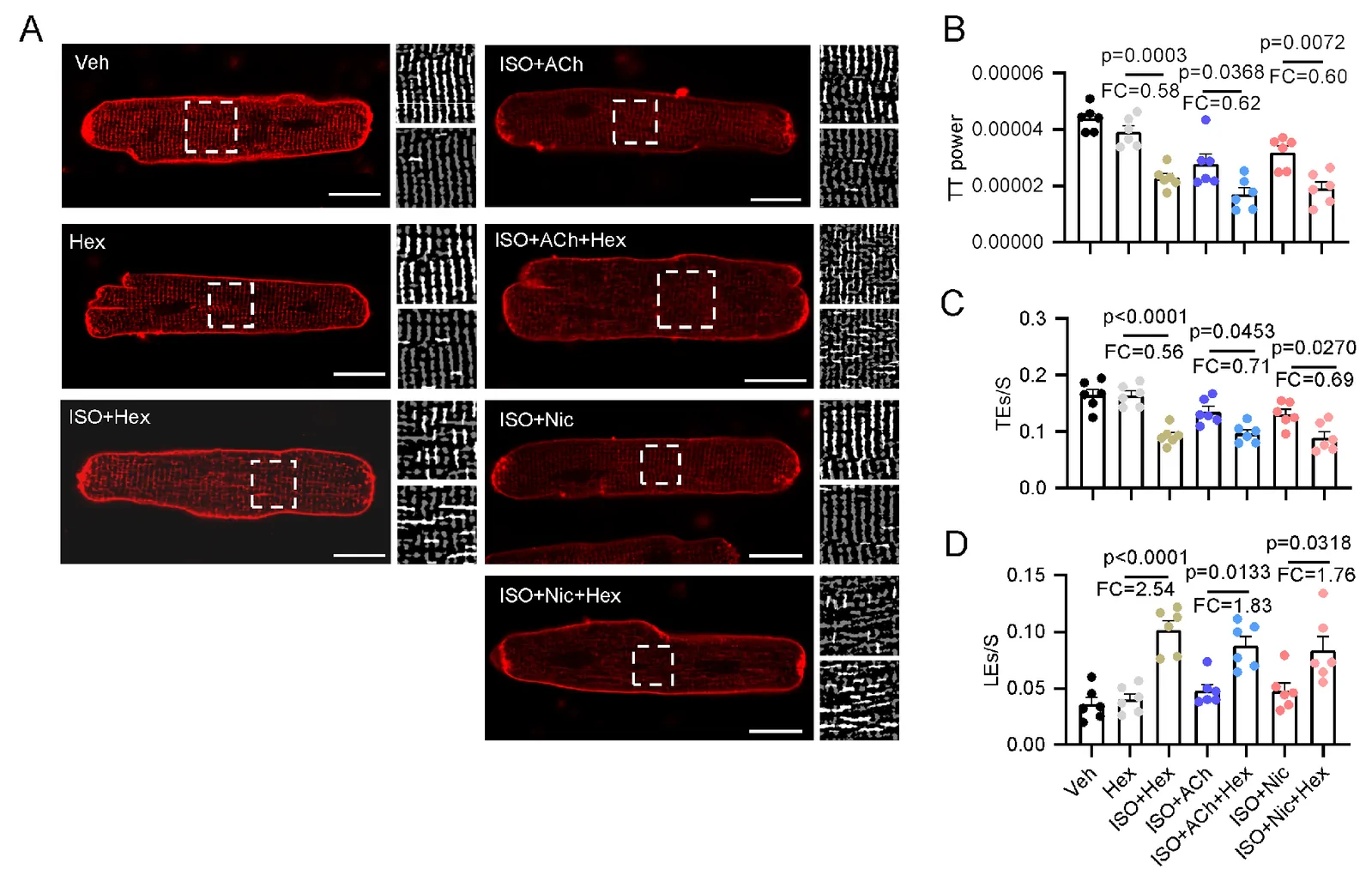
Effects of hexamethonium bromide (Hex) on ISO-induced TT changes in the presence of ACh or Nic. (**A**) Representative images of CellMask Deep Red-stained adult rat ventricular cardiomyocytes as shown in Figure 1A. ISO + ACh + Hex: co-treatment with isoproterenol (100 nM), acetylcholine, and hexamethonium (10 μM each) for 48 h, ISO + Nic + Hex: co-treatment with isoproterenol (100 nM), nicotine, and hexamethonium (10 μM each) for 48 h. Scale bar = 20 μm. (**B**–**D**) Quantitative analysis of the TT network structure as with metrics corresponding to those in Figure 1B–D. Data are presented as mean ± SEM. N = 6 rats (5 males and 1 female). Statistical significance was assessed by a RM one-way ANOVA with Dunnett’s post hoc test. For clarity, only *p* values for the pre-planned comparisons of interest are indicated above each figure. A complete table of exact *p* values and 95% confidence intervals can be found in Supplementary Table S1.

Thus, despite inducing baseline TT remodeling when administered alone, nAChR activation during ISO exposure coincided with less severe TT disruption. To verify the specificity of this effect, we applied the nAChR antagonist hexamethonium bromide (Hex). Under these conditions, Hex appeared to attenuate the effects of ACh and Nic on ISO-induced TT damage (Figure 4A–D), suggesting that nAChRs mediate this protective cellular response.

## 3. Discussion

In the present study, we demonstrate that TT structure is directly regulated through modulation of both adrenergic and cholinergic receptors, particularly the nicotinic cholinergic receptors. Co-treatment with either acetylcholine or nicotine appeared to modulate T-tubule orientation and density, with some attenuation of ISO-induced structural disruption and cellular hypertrophy also observed. These fundings are preliminary and require further validation.

A possible explanation is that activation of adrenergic and nicotinic cholinergic receptors alone alters cardiomyocyte Ca^2+^ homeostasis [18,19]—one via cAMP-mediated phosphorylation of Ca^2+^-handling proteins (e.g., ryanodine receptor 2, RyR2, L-type calcium channel, LCC), and the other by directly modulating cytosolic Ca^2+^ concentration as a cation channel. Because nAChR activation has also been reported to activate calpain-1 in PC12 cells [20], a parallel mechanism may exist in cardiomyocytes, where it could drive calpain-dependent cleavage of JPH2 and subsequent TTs remodeling, such as ISO-induced structural damage.

The paradoxical protection of TTs during ISO and nicotine co-treatment might plausibly be explained by a proposed bidirectional negative crosstalk between their downstream signaling pathways. On one hand, ISO could activate the β-adrenergic receptor/cAMP/cAMP-dependent protein kinase (PKA) pathway to suppress nicotine’s toxicity. Previous studies have established that PKA directly phosphorylates nAChR subunits in muscle cells [21] as well as in neurons [22]. This phosphorylation not only regulates the assembly and surface expression levels of the receptor but, crucially, accelerates the rapid desensitization of nAChR [23,24,25]. Consequently, we hypothesize that this PKA-driven desensitization may effectively limit nicotine-induced Ca^2+^ signaling. On the other hand, nicotine appears to reciprocally block ISO-induced TTs disruption. Based on previous findings in neuronal models, nAChR-triggered Ca^2+^ influx stimulates the phosphatase calcineurin [26]. This activated calcineurin might act as a molecular counterweight to PKA, potentially dampening subsequent L-type calcium channel activity and antagonizing ISO-mediated hyperphosphorylation. Ultimately, this putative kinase-phosphatase (PKA vs. calcineurin) antagonism would theoretically maintain intracellular Ca^2+^ below the critical threshold for calpain activation, thereby preventing structural cleavage and preserving TTs integrity. Another factor, albeit less probable, also warrants consideration: the activation of the nAChR-mediated phosphoinositide 3-kinase (PI3K)/AKT signaling pathway, a master regulator known to mediate profound anti-apoptotic and survival effects in tumors [27,28], neurons, and cell lines [29], may also contribute to the nAChR-mediated effect.

From a clinical and translational perspective, although very preliminary, our in vitro data provide a possible cellular mechanism for identifying a potential process by which cholinergic signaling may modulate cardiomyocyte TT integrity under stress, specifically at the level of nicotinic receptor-mediated survival signaling, may be relevant to the cardioprotective effect. Nicotine, existing at high prolonged concentrations (25–400 nM) in the blood of smokers [30], has historically been linked globally to arrhythmias [31] and cardiovascular mortality [32,33,34]. However, our findings suggest that high ambient nicotine levels might paradoxically pre-desensitize nAChRs and trigger intracellular survival cascades, potentially preserving crucial excitation-contraction coupling structures against the acute catecholamine storm during severe cardiac stress.

Ultimately, the multifaceted role of cardiac nAChRs and the interpretation with β-adrenergic receptor requires further investigation. To determine whether the mutual antagonism between PKA and calcineurin can stably prevent TTs disruption caused by individual treatments through myocardial Ca^2+^ regulation and reduction of calpain-mediated cleavage, higher spatiotemporal resolution to synchronously evaluate the crosstalk and spatial distribution mechanisms of competing downstream Ca^2+^ signals upon the co-activation of two receptors is necessary. Furthermore, while our current findings using Hex provide preliminary evidence for nAChR involvement, future studies utilizing subtype-selective antagonists (e.g., α-bungarotoxin, α-BGT and methyllycaconitine, MLA) or α7 knockout/knockdown models are needed to identify the specific receptor subtype. In addition, future studies are warranted to employ three-dimensional electron microscopy approaches, such as focused ion beam-scanning electron microscope (FIB-SEM), to comprehensively assess the ultrastructural changes in treated cardiomyocytes, as conventional two-dimensional imaging cannot capture the full spatial complexity of TTs remodeling process. In addition, mechanistic investigations should be conducted on potential downstream target proteins, such as the expression and cleavage of JPH2, as well as on the signaling transduction processes using specific inhibitors such as H-89 (for PKA) [35,36] and cyclosporin A (for calcineurin) [37,38], to identify possible causal relationships and verify the validity of the hypothesis. Furthermore, while nAChR-mediated rescue prevents ISO-induced TT fragmentation, we interpret this as preserving structural integrity rather than a direct anti-hypertrophic effect, given the spontaneous dedifferentiation of cultured adult cardiomyocytes. In vivo studies utilizing definitive molecular markers are essential to validate these mechanisms and clarify the therapeutic potential of cardiac nAChR modulation in the future.

The present findings should be interpreted as correlative rather than as direct evidence of functional protection. Because our experimental design was not optimized to assess complex pharmacological interactions, evaluating key functional indicators of excitation-contraction (EC) coupling at the cellular level, such as spatial Ca^2+^ transient/spark dynamics, L-type current (I_Ca,L_), and action potential duration (APD), would be highly beneficial. Ultimately, alongside these single-cell assessments, future in vivo investigations are necessary to determine whether this structural rescue translates into functional cardiac improvements. While sex hormones are known to influence cardiovascular pathophysiology [39], studies specifically addressing sex-dependent variations in T-tubule architecture and nAChR expression remain limited. Moreover, sex differences in β-adrenergic signaling have been documented [40]. Although our sensitivity analysis confirmed that pooling sexes (inclusion of female data) did not alter our conclusions, this remains a limitation. Therefore, future research should include both male and female cohorts to systematically determine whether crosstalk between nAChR and β-adrenergic signaling, and its consequent cardioprotective efficacy, depends on sex. Such future investigations will be essential to fully understand the translational implications of our findings.

## 4. Materials and Methods

### 4.1. Animals

The animal handling procedures (No. LSC-WangSQ-1) were approved by the Institutional Animal Care and Use Committee (IACUC) at Peking University, accredited by AAALAC International. The animal experiments conform to the Guide for Care and Use of Laboratory Animals published by the US National Institutes of Health (NIH Publication No. 85-23, revised 2011). Sprague-Dawley (SD) rats were purchased from Beijing Vital River Laboratory Animal Technology Co., Ltd. (Beijing, China). The rats were housed in a specific pathogen-free barrier environment and were kept on a 12 h light–dark cycle at 21–23 °C. Eight-week-old (about 200 g) rats were randomly used in the experiments unless criteria were met for euthanasia, including hunching, ruffled fur, labored breathing, low body temperature, and lack of mobility. Both female and male animals were used for this study, and no rats were excluded from the experiments based on these criteria in our study.

### 4.2. Cardiomyocytes Preparation

Ventricular cardiac myocytes were enzymatically isolated as described previously [3,18]. Rat hearts were rapidly removed under isoflurane (Hebei Yipin, Shijiazhuang, China) anesthesia and mounted on a Langendorff apparatus. The heart was then perfused for 5 min at 37 °C with a Ca^2+^-free Tyrode’s solution containing (in mM) 135 NaCl, 4 KCl, 1 MgCl_2_, 1.2 NaH_2_PO_4_, 11.9 NaHCO_3_, 10 glucose, and 10 taurine saturated with 95% O_2_ and 5%CO_2_. Type II collagenase (0.7 mg/mL) (Worthington Biochemical, Lakewood, NJ, USA) was then added to the perfusate. After 15–20 min, the heart was taken down, and the ventricle was cut into small pieces. Finally, cardiomyocytes were centrifuged and resuspended in Tyrode’s solution containing 1 mM Ca^2+^.

### 4.3. Confocal Imaging

An inverted confocal microscope (Zeiss LSM 710, Oberkochen, Germany) with a 40×, 1.3 NA oil-immersion objective was used for imaging. For membrane labeling, freshly isolated rat cardiomyocytes were cultured with the indicated drugs for 48 h in an incubator. All pharmacological agents were administered simultaneously (co-treatment). Subsequently, the cells were incubated with CellMask Deep Red (Thermo Fisher Scientific, Eugene, OR, USA) for 10 min at room temperature. Fluorescence was measured with excitation at 647 nm and emission at >660 nm. For the cardiomyocytes isolated from each rat, the culture dishes were randomly allocated to the control and treatment groups, and to minimize potential confounders in this in vitro setting, treatments and measurements were performed in a randomized and balanced order. For each dish, five fields of view were randomly selected from different regions. Based on the CellMask signal, we selected only viable, rod-shaped cardiomyocytes for imaging and subsequent T-tubule analysis, excluding spherical cells. Analyses were performed in a blinded manner with an IDL program. Briefly, TT power (derived from Fast Fourier Transform (FFT) analysis and reflecting the regularity of the TT network), the number of transverse elements per ROI (TEs/S), and the number of longitudinal elements per ROI (LEs/S) were quantified as described previously [3,41,42].

### 4.4. Statistics

Statistical analysis was performed using GraphPad Prism 9.5. No experiment-wide/across-test correction was performed. For multiple group comparisons, Tukey’s or Dunnett’s post hoc correction was applied. All data are mean ± SEM as indicated. The statistical methods are also specified in the figure legends, and exact *p*-values and 95% confidence intervals are provided in Supplementary Table S1.

## 5. Conclusions

In conclusion, although prolonged sympathetic stress (ISO) severely disrupts TTs architecture and cellular morphology, co-activation of nAChRs by acetylcholine or nicotine was associated with less pronounced damage under these conditions. Despite inducing mild TTs remodeling when administered alone, nAChR activation may act as a potential antagonist against ISO-induced structural deterioration.

## Data Availability

The raw data supporting the conclusions of this article will be made available by the authors on request.

## Supplementary Materials

The following supporting information can be downloaded at: https://www.mdpi.com/article/10.3390/ijms27219597/s1.

## Abbreviations

The following abbreviations are used in this manuscript:

TT, TTs	Transverse tubule, Transverse tubules
BIN1	Bridging integrator 1
JPH2	Junctophilin 2
ACh	Acetylcholine
ISO	Isoproterenol
Nic	Nicotine
Xano	Xanomeline
Hex	Hexamethonium bromide
RyR2	Ryanodine receptor 2
LCC	L-type calcium channel
PKA	cAMP-dependent protein kinase
FIB-SEM	Focused ion beam-scanning electron microscope
EC coupling	Excitation-contraction coupling
APD	Action potential duration
α-BGT	α-Bungarotoxin
MLA	Methyllycaconitine
